# COVID-19 Genetic Variants and Their Potential Impact in Vaccine Development

**DOI:** 10.3390/microorganisms10030598

**Published:** 2022-03-10

**Authors:** Giau Van Vo, Eva Bagyinszky, Seong Soo A. An

**Affiliations:** 1 Department of Biomedical Engineering, School of Medicine, Vietnam National University Ho Chi Minh City (VNU-HCM), Ho Chi Minh City 70000, Vietnam; vvgiau@medvnu.edu.vn; 2 Research Center for Genetics and Reproductive Health (CGRH), School of Medicine, Vietnam National University, Ho Chi Minh City (VNU-HCM), Ho Chi Minh City 70000, Vietnam; 3 Vietnam National University Ho Chi Minh City (VNU-HCM), Ho Chi Minh City 70000, Vietnam; 4 Graduate School of Environment Department of Industrial and Environmental Engineering, Gachon University, Seongnam 13120, Korea; 5 Department of Bionano Technology, Gachon University, Seongnam 13120, Korea

**Keywords:** SARS-CoV-2, COVID-19, Omicron, VOCs, vaccine development

## Abstract

In the two years since the SARS-CoV-2 pandemic started, it has caused over 5 million deaths and 400 million infected cases, and the world continues to be on high alert for COVID-19. Among the variants of interest and concern of SARS-CoV-2, the current Omicron (B.1.1.529) and stealth Omicron (BA.2) raised serious concerns due to rapid rates of infection caused by numerous mutations in the spike protein, which could escape from the antibody-mediated neutralization and increase the risk of reinfections. Hence, this work aims to describe the most relevant mutations in the SARS-CoV-2 spike protein, discuss vaccine against variant of concerns, describe rare adverse events after COVID-19 vaccination, introduce the most available promising COVID-19 vaccine candidates, and provide few perspectives of the future variants.

## 1. Introduction

SARS-CoV-2 (COVID19) is a novel virus, which emerged from Wuhan in 2019 (China) and became a major public, social, governmental and research issue all around the world. In healthy people, the virus can cause disease symptoms similar to flu (fatigue, migraine, diarrhea, fever, coughing, chills, muscle aches, shortness of breath, loss of smelling and tasting) with a high rate of death depending on the mutations. However, in the elderly and people with disease/illness comorbidities, the virus could further develop serious symptoms, such as failures of the respiratory system, eyesight, heart and/or kidney functions, resulting in inflammation and sepsis [1,2]. Other minor presentations were the coloration of skin, hair loss and chest pain [2]. As a zoonotic virus, it could transmit rapidly from person to person and animal to human, especially in case of close contact [2].

**Figure 1:**
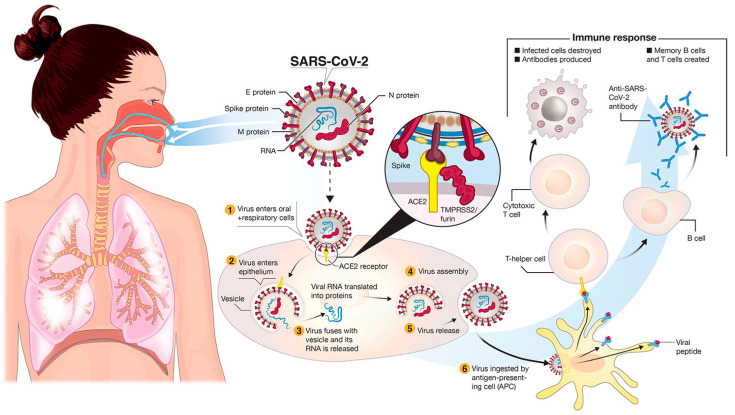
Transmission and life cycle of SARS-CoV-2 causing COVID-19. SARS-CoV-2 is transmitted via respiratory droplets of infected cases to oral and respiratory mucosal cells [6].

Coronaviruses belong to the Coronaviridae family, order of Nidovirales. Four different subfamilies could be distinguished in coronavirus: Alpha, Beta, Gamma and Delta coronavirus. Coronavirus genomes contains a single-stranded positive RNA with a cap structure the 5′ end and a PolyA-tail at the 3′ end. Genome of SARS-CoV-2 encoded 16 nsps, 4 structural and 6 accessory proteins [3]. Coronaviruses were reported to have at least six open reading frames (ORFs), from which the first frame (ORF1a/1b) was the longest (2/3 of entire genome). ORF1a/1b encoded 16 (15 in Gamma coronavirus) nonstructural proteins (nsps), which formed the replication–transcription complex (RTC). Nsps proteins could have different functions, such as inducing cellular mRNA degradation, helicase, ribonuclease, viral RNA transcription, and the translation immune evasion. The remaining five ORFs encoded many structure proteins, including the spike (S), membrane (M), envelope (E), and nucleocapsid (N) proteins, and additional accessory proteins [3,4,5]. The spike formed a trimer and interacted with angiotensin-converting enzyme 2 (ACE2), initiating the viral infection. The E-protein could create an ion channel, which facilitated the viral material to enter into the host cell, and an N protein initiated the binding of the host protein to host DNA, while the M protein was important in viral morphogenesis. Accessory proteins included 3a, 6, 7a,7b, 8 and 9b, which were suggested to regulate the immunity of host [3] as reveals in Figure 1.

The current issue with SARS-CoV-2 is the rapid alteration of its pathogenic potentials, which could be a significant challenge for the development of vaccines and drugs. The virus genome could mutate into different variants from insertions, deletions and alterations, which would be important for a potential forecast for accessing and developing protective vaccines/drugs against the different upcoming variants of SARS-CoV-2 [7]. This review summarized all variants of SARS-CoV-2 that could be related to disease cases, their symptoms, and how they would react to vaccinations and treatments.

## 2. Genetic Variants of SARS-CoV-2

The majority of RNA viruses have high rates of mutation. Until SARS-CoV-2, coronaviruses were thought to have a lower rate of mutation than other RNA viruses. The genomes of coronaviruses contain a responsible repair enzyme to correct replication errors. Even though the fate of the variants in the viral genome may be determined by natural selection, several variants may escape from the immune system and result in pandemic transmission. On the other hand, other variants may weaken viral fitness regarding the host immune system, reducing its virulence [8,9]. Since it would be difficult to define the effect of natural selection in terms of different virus variants, sequencing updated variants or mutations in the SARS-CoV-2 genome could be essential. Importantly, the differences, “variant” and “mutation”, should be clearly defined. Mutation means the amino acid exchange (nonsynonymous or missense) in the spike glycoprotein, while other changes in nucleotide (synonymous or non-missense) may be defined as variants [8,9]. The first reported variant of the SARS-CoV-2 genome in the spike protein was the D614G (A23403G), which spread rapidly and became the first globally prevalent strain [10]. This mutation was identified in the Wuhan reference strain in March 2020 [11]. This mutation may co-exist with other variants: a C-to-T exchange in the 5′ UTR region (C241T); silent C-to-T variant at position 3037 (A23403G); and with a missense mutation in the viral polymerase region, P323L (C14408T) [12]. These four mutations could combine as a haplotype related to the Wuhan strains, and this this haplotype was connected to in the dominant form of the disease. Interestingly, this P323L variant may be missing in certain strains from China and Germany [12]. Strains with G614 were found to spread more rapidly than D614. A plaque assay of viral strains found that SARS-CoV-2 isolates with G614 presented with a higher virus titer than D614 isolates. In addition, the formation of syncytium and the cleavage of the S-protein was accelerated in G614 isolates in. The G614 isolates may enhance viral transmission and infection through the enhanced syncytium formation by furin-dependent S-cleavage [10]. High temperatures (33, 37 and 42 °C) may reduce the infection rate in both viruses with G614 and D614. G614 presented a stronger infection rate than D614 at all temperatures, which may provide additional proof of its stability [13]. A comparison of viral isolates with D614 and G614 spike proteins revealed that G-614 virus isolates could bind angiotensin-converting enzyme 2 (ACE2) with a higher efficacy, even after refrigeration or freezing. These findings suggested that SARS-CoV-2 with G614 may remain stable at a low temperature, which could be responsible for its rapid transmissions with high infectivity [14]. An experiment where hamsters were infected with SARS-CoV-2 with G614 variant suggested increased viral loads in the upper respiratory system (nasal area and trachea, but not in lungs). Furthermore, the replication rate of the virus with G614 was higher in human lung Culu3 cells [13].

## 3. Virus Variants of Concern (VOCs) and Former VOCs

**Figure 2:**
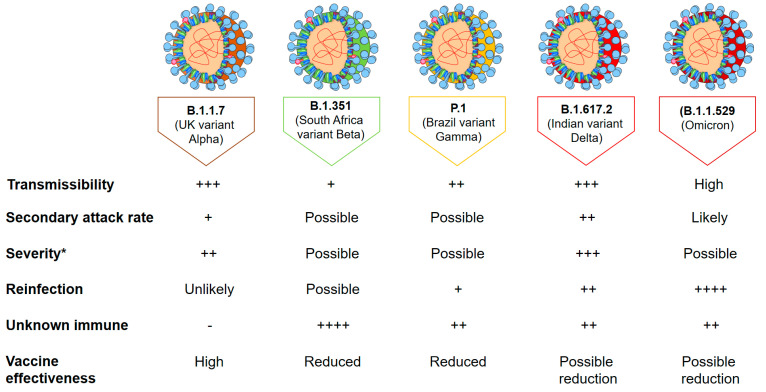
Properties of SARS-CoV-2 variants of concern. * Severity is determined by an increased risk of hospitalization and increased risk of mortality. The +…++++ means the degree of disease severity, transmissibility etc.

Variants of concern (VOCs) in SARS-CoV-2 were defined by an enhanced transmissibility or infectivity and were suggested to have a strong impact on public health. These VOC variants usually focused on several mutations (except from D614G) in spike proteins, which could affect disease severity and the reaction of vaccines and therapies. These variants may facilitate the rapid spreading of the virus and its ability to escape the immune system. Five SARS-CoV-2 variants categorized described as VOCs: Alpha, Beta, Gamma, Delta, Omicron variants. Unexpectedly, the Alpha variant was drastically reduced due to the spreading of the Delta variant, which was also overwhelmed by the appearance of Omicron variants, in which the “former VOC” was de-escalated as the newer variant with a higher infectivity (Figure 2).

The VOC 202012/01 (lineage B.1.1.7-Alpha variant; British mutation) variant emerged in the second half of 2020 in Southern England. In the Alpha variant, the mutation rate was high, and it was transmitted rapidly, especially in patients with immunodeficiency or immunosuppression: https://virological.org/t/preliminary-genomic-characterisation-of-an-emergent-sars-cov-2-lineage-in-the-uk-defined-by-a-novel-set-of-spike-mutations/563, accessed on 29 December 2021. Seventeen mutations were discovered in the VOC 202012/01 variant, which included 14 missense mutations and 3 indels. Among these, three were suggested to be significant: N501Y, P681H and ∆H69/∆V70, whereas N501Y could be the key residue in pathogenicity through an enhanced binding to the human ACE2 receptor. The second significant variant was P681H near the furin cleavage site. Furthermore, the deletion of ∆H69/∆V70 in the spike region could be involved in the viral escape from the immune system. An additional potentially interesting mutation could be Q27X, resulting in the deletion of 382 amino acids in the viral genome, which was originally isolated from Singapore in March 2020. This Q27X variant itself was associated with minder disease symptoms in comparison with the original SARS-CoV-2. Even though the loss of ORF8 may result in a minor effect on viral replication, these studies suggested the impartiality of ORF8 to modulate the immune response of host or gene expressions [15]. However, this variant spread rapidly all over England with a higher rate of transmission, unfortunately due to a higher number of social interactions. This Alpha variant also spread globally with similarly high transmission rates in Europe and the USA [16]. The clinical outcomes of VOC 202012/01 had similar outcomes to the other SARS-CoV-2 variants with accelerated rates of transmission [17]. The mortality of the Alpha variant depended on the age of patients. Patients above 70 years of age had a higher risk of hospitalization and mortality than younger patients [18]. The majority of children who were infected by the Alpha variant presented with fever, rhinitis and cough, and their symptoms were mild [19].

The Beta variant (B.1.351 or GH501Y.V2) was a South African mutation that emerged from South Africa in October 2020. Starting in a metropolitan area (Nelson Mandela Bay), it spread rapidly all around the country and became the dominant strain in several provinces within a few weeks. The Beta variant carried nine missense mutations: L18F, D80A, D215G, R246I, K417N, E484K, N501Y, D614G, and A701V. Among them, K417N, E484K and N501Y were located in the key binding sites in the receptor-binding domain. E484K and N500Y strains could enhance virus binding to ACE2, whereas the combination of two mutations could further accelerate virus–receptor interactions [20]. Among them, K417N also appeared in the Beta variant. Structure predictions revealed that these three mutations could result in significant changes to the receptor-binding domain of the spike protein, resulting in stronger ACE2 binding [21]. Patients carrying variants in SARS-CoV-2 residue 484 (E484K, E484Q or E484P) showed a reduced ability to neutralize the infection by polyclonal antibodies. Mutations in residue 484 were found to decrease the neutralization titer against the spikes of virus by 35–40 times [22]. They could also act as “escape mutations”, since they may be able to avoid the immune response of the human body [23]. Humanized ACE2 mice (hACE2) were infected with the Beta variant. The mice experienced significant weight loss in the first 3 days of infection. Lung injury started at 1-day post-infection and became worse on the third day. Damage was experienced in bronchiolar epithelial cells, and the alveolar septa was thickened. Hemorrhage and macrophages infiltration was also observed in mice. In addition, cytokine levels (such as IL-1Alpha, IL-2, IL-5, IL-6, IL-17A) were elevated in sera. Recovery started 7 days after infection, and no death was observed [24].

The P1 (B.1.1.28; GR/501Y.V3, Brazilian mutation) or Gamma variant emerged from Brazil, and it was first recognized at the end of 2020. Brazil was severely affected by SARS-CoV-2, especially the northern regions around Amazonas (Manaus). Manaus is a crowded region of Amazonas, and the first case of acute respiratory syndrome was observed in spring 2020 [25]. The virus was associated with a rapid spread all around Brazil. Almost half the population of Manaus had detectable antibodies (IgG) after the epidemic peak of this virus. The estimated attack rate was 66% in June 2020 in the Manaus region and 76% in October 2020. In Sao Paulo, the estimated attack rate was lower in October 2020 [25]. In early 2021, this variant appeared in Japan, spread by those traveling from the Amazonas region. Seventeen missense mutations appeared in the Gamma variant, and among them, three key mutations were identified in the spike region: K417T, E484K, and N501Y. Aside from these mutations, three deletions, four silent variants and a four-base insertion were also observed in P1 variant [26]. The three key variants were located in the RBD region of spike protein. The N501Y and E484K were already detected in the Beta variant, and were suggested to enhance virus–ACE2 interactions. Furthermore, the Gamma variant was associated with a low degree of antibody neutralization [26]. Structure predictions revealed that N500Y may play a significant role in virus binding to ACE2. The K417T and E484K may enhance viral expression and enable the virus to escape from the human immune system [27]. The Gamma variant revealed a 1.7- to 2.4-fold higher degree of transmission compared to the non-P.1 variants [26].

The Delta variant of SARS-CoV-2 (B.1.617, Indian mutation) emerged from India in late 2020 and spread all around the world in more than 100 countries. In early 2021, the initial SARS-CoV-2 variant spread in India, called the “double mutant”, and carried two spike mutations: L452R and E484Q. Later, this variant went out through modifications, and additional mutations appeared in the sequence clusters [28]. The Delta variant may not be homogenous, since several mutations were found in the spike regions of Delta variants, including T19R, Δ157–158, L452R, T478K, D614G, P681R, and D950N. The pathogenic nature of these strains may be different depending on the variants. They may affect the immune response of the host, especially the mutations, located in the RBD domain (L452R, T478K).

The P681R variant is located in the S1–S2 cleavage site, and may result in elevated viral loads and transmission rates [29,30]. The mutations located in the N-terminal region of spike protein could also enhance viral resistance to antibodies and its escape from host immunity [31]. Variants may also affect the immune response of the host, especially if they carry variants in the antigen-receptor-binding area (at residue 452 or 478). The Delta variant was associated with larger RNA loads and a greater transmissibility, no matter the vaccination status. The reproductive number of Delta variants was estimated to be higher (5–8 fold) compared to the previously described virus variants. The mutations in the spike region could enable efficient viral entry into host. Additionally, the enhanced syncytium formation may result in higher viral loads and severe disease symptoms. The incubation time of virus became lower, and the risk of hospitalization increased, including in the younger individuals [28,32,33,34,35,36].

In November 2021, a novel variant called the Omicron variant (or B.1.1.529) emerged from the southern region of Africa [37]. This variant spread quickly, since it appeared first among African countries but was recently found in the USA, Europe and Asia too [38,39]. Several mutations were identified in Omicron variants, which may impact the disease severity and viral transmission. At least thirty mutations were identified in the spike protein, and from them, 15 were found in the receptor-binding domain: G339D, S371L, S373P, S375F, K417N, N440K, G446S, S477N, T478K, E484A, Q493R, G496S, Q498R, N501Y, Y505H, T547K, D614G, H655Y, N679K, P681H, N764K, D796Y, N856K, Q954H, N969K, and L981F. The L452R variant was not observed in the Omicron variant [39]. The majority of these variants were found to be unique to the Omicron variant, since they did not appear in the previously described SARS-CoV-2 variants. Among them, N501Y, S317L, S373P, S375F, E484A, Q498R and T478R were found to increase the viral interaction with the ACE2 protein. E484A and Y505H could reduce the contact between the virus and host antibodies and enhance the viral ability to escape from the host immune system. K417N could also impact the conformation changes in the spike protein and immune escape. Additional mutations also appeared in other viral genes (ORF1a, ORF1b), which were prevalent in Omicron strains, and the majority of them were unique to the Omicron variant. Two of these mutations (ORF1a T492I and ORF1b: P314L or nsp12: P323L) were also present in Delta variants, and P323L co-evolved with the D614G mutation [38]. Due to these mutations, the Omicron variant binds ACE2 with a higher affinity compared to Delta variant. Additionally, its transmission was also found to be higher than the previously described viral strains [40]. Phylogenetic studies suggested that the Omicron variant may be closely related to the Gamma variant [38]. Currently, the WHO is monitoring the Omicron variant, since it carries several mutations in the spike protein, and the related cases have increased rapidly. Due to its current status, there are concerns of disease severity and transmissibility, since the mutations may be related to a higher virulence [41]. Further studies are needed on whether the Omicron variant could be neutralized by different vaccines. The Omicron variant could potentially escape from the immune system, but the two doses of vaccines, consisting of an inactivated virion and homologous booster shot, may improve viral neutralization [42]. The Omicron variant was described to be highly infective, and it may attack the upper respiratory airways more efficiently than previous variants. Animal experiments also suggested that the Omicron variant could result in milder disease symptoms in both mice and hamsters [43]. Different from the Delta variant, the Omicron variant may replicate more rapidly in the bronchus, but it was less effective in infecting the lung tissues [44].

## 4. Variants of Interest (VOIs) and Former VOIs

Aside from VOCs, additional variants were described in SARS-CoV-2, which carried genetic mutations, potentially impacting viral transmission, disease severity and therapies. These variants may be at risk of becoming VOCs, since they may result in rapid transmission. Similar to VOCs, VOIs may be downgraded into “former VOI”, when they may no longer affect public health. Currently, the Mu and Lambda variants are categorized as VOIs along with other former VOIs, such as the Epsilon variant and Kappa variant.

When B.1.427/B.1.429 (clade 21C), or the Epsilon variant, started to spread from California, this virus resulted in a massive growth in infection, causing an outbreak in the USA. In Californian strains, L452R was the only mutation in the viral spike regions. The Epsilon variants could harbor the following mutations: B.1.427: L452R, D614G; B.1.429: S13I, W152C, L452R, and D614G. L452R was associated with a strong positive selection of the virus. Another variant, L452Q, known as the Lambda variant, was reported [45], which may enhance the stability of the spike protein, as well as viral infectivity and replication rates. Additionally, L452R also increased the binding affinity towards the ACE2 receptor and was able to escape from the immune system of the host, especially the HLA-A24-restricted cellular immunity. L452 loci seemed to further increase the ability of viral fusion [46]. The advantage of this was that the Epsilon variant may not be associated with a high degree of hospitalization and ICU administration. Its prevalence has decreased since April 2021. Even though the symptoms of this variant seemed to be severe, vaccines seemed to be effective. However, the Epsilon variant should not be ignored, as it could be possibly present a higher virulence in certain groups of patients [47].

The Lambda variant (C37) was first identified in December 2020 in Peru and spread quickly throughout other South American countries (Argentina and Chile). It was suggested to have a higher transmissibility than the original Wuhan strain. The Lambda variant also appeared in Europe (Spain, Germany, and France), containing seven mutations in the spike region: G75V, T76I, D253N, L452Q, F490S, D614G, and T859N. Additionally, the Lambda variant also contained a deletion (del246 to 252) in the N-terminal domain and a deletion (del3675 to 3677) in the ORF1A gene. Among the spike regions, two mutations, L452Q and F490S, were located in the RBD area. L452Q was a specific variant in the Lambda variant [48]. Based on the findings on L452R and L452Q, they may also enhance the affinity to the ACE2 receptor. Furthermore, F490S could also increase viral infectivity when L452Q was present, but without L452Q, it may not impact infectivity [49,50]. Viral transmission was accelerated in the spring of 2021, reaching its peak between April and June. After 31 epidemiological weeks, the cases of the Lambda variant started to reduce and were surpassed by the Gamma variant [51].

The Kappa variant (B.1.617.1) emerged from India, which was suggested to be the forerunner of the highly transmissible Delta variant. The Kappa variant harbored several mutations in the spike region, including T95I, G142D, E154K, L452R, E484Q, D614G, P681R, and Q1071H [52]. Among them, L452R and E484Q were located in RBD, which were also observed in other SARS-CoV-2 variants. However, in the Kappa variant, these two mutations co-existed for the first time with a strong reduction in the affinity between viral RBD and monoclonal antibodies [53]. The P681R mutation was located near the furin cleavage site, which may influence viral entry into the host cell. Among non-spike proteins, R203M was found in the N-protein, which could impact viral assembly [54]. The Kappa variant may have a higher tendency of escaping from vaccine-induced immunity than the Delta variant. Nonetheless, the Delta variant became more dominant due to its higher transmissibility, higher replication rate and larger viral loads in upper respiratory system [52].

The Eta variant (B.1.525) emerged from the USA (New York) at the end of 2020, followed by reports of at least 50 countries all around the world. This variant is currently categorized as a variant of interest due to its mutations for a stronger virulence and transmissibility: A67V; deletion of residues 69/70; deletion of residue 144; E484K; D614G; Q677H; and F888L. The Q677H mutation may be involved in modulating transmissions, and the deletion of residue 144 may enhance its escape from the immune system. F888L in the S2 domain of the spike protein may also impact the virulence of SARS-CoV-2 [55].

The Iota (B.1.526) variant also emerged from the USA (New York) and contained several spike variants: L5F, T95I, D253G, S477N, L452R, E484K, D614G, and A701V. The Iota variant seemed to have three sub-lineages: B.1.526-E484K, the B.1.526-S477N or B.1.526-L452R, depending on the key mutations in the non-spike mutations; T85I, L438P, residue 106-108 deletion in ORF1a, P323L and Q88H in ORF1b; Q57H in ORF3a; and P199L and M234I in N gene. The Iota variant spread rapidly among USA and in other countries. It was reported to be associated with antibody resistance, especially the B.1.526-E484K lineage. However, its transmission rate was recently overtaken after the rise of the Alpha variant [56].

The Mu variant (B.1.621) emerged from Columbia in early 2021 and was detected in more than 50 countries by late summer of 2021. The majority of Mu variants carried different mutations. In the N-terminal domain, they carried T95I and YY144-145TSN mutations. Several mutations were found in the spike protein, including 69/70 deletion, Y144T, Y145S, ins146N, R346K, E484K, N501Y, D614G, P681H, and D950N [57]. The combination of 69/70 deletion, E484K and N501Y may highly impact antibody neutralization. Additionally, the asparagine insertion at residue 146 may impact the viral S1 closed–open conformation, and ACE2 binding [58]. The Mu variant was also suggested to have a possible immune escape ability, since it was suggested to be strongly resistant to antibodies, and may affect the vaccine efficacy [13,59]. The Mu variant was not considered as a variant of concern, since its characteristics (virulence, resistance to vaccination, immune escape) have not yet been fully researched [60].

The P.3 (or Theta) variant emerged from the Philippines in March 2021. A phylogenetic analysis revealed that it may be closely related to the B.1.1.7 variant. In the P.3 variant, 14 missense mutations were identified with 4 key variants in the spike region—E484K, N501Y, D614G, and P681H—which could contribute to a stronger infectivity and ACE2 binding. Additional variants appeared in the C-terminal spike region: E1092K, H1101Y, and V1176F. The deletion of residue 141–143 was also found in P.3 variant, which may be important for immune escape mechanisms [61]: https://pgc.up.edu.ph/sars-cov-2-bulletin-no-7/, accessed on 30 December 2021. In July 2021, cases of the P.3 variant were reported to be reduced, and thus the variant was removed from the list of VOI variants. Interestingly, recent studies found a similar virus among service workers (oil ring) in Louisiana. The Louisiana -P.3 viruses carried an additional spike variant, Q1180H, and the deletion of residue 141–143 was noticed as missing in some patients. Fortunately, this variant was identified only once, and workers were quarantined. Even though no additional cases were identified in other employees and other regions, P.3 variant should be monitored [62].

The P.2 (or zeta) variant was reported in Brazil and was a descendant of the B.1.1.28 strain. P.2 carried a few additional variants in addition to B.1.1.28, including a C100U exchange in 5′ UTR region and C29754U exchange in the 3′ UTR region. This variant carried the E484K variant, without the N501Y or K417T [63] variants. Initially categorized as VOI, the World Health organization removed P.2 from the list of VOIs: https://www.who.int/en/activities/tracking-SARS-CoV-2-variants/, accessed on 10 January 2022.

The AY4.2, called “Delta Plus” variant, were detected in the UK in October 2021 with possible Indian origin. It belonged to the original sub-lineage of B.1.617. The Delta Plus variant seemed to present a 20% higher virulence than the original Delta variant. Five key mutations were detected in the spike region of “Delta Plus”, which were more prominent than those in the Delta variant, including T95I, A222V, Y145H, G142D, R158G, and K417N [64]. Y145H and A222V mutations may also have an impact on viral functions, such as immune escape and a higher transmissibility [65]. AY.4.2 was found to have slightly higher transmissibility than the original Delta variant. Fortunately, it did not have a higher immune escape ability than the original Delta variant regarding disease symptom severity [66,67]. It may be possible that treatments and vaccinations are equally effective against both Delta and Delta Plus variants [67].

**Figure 3:**
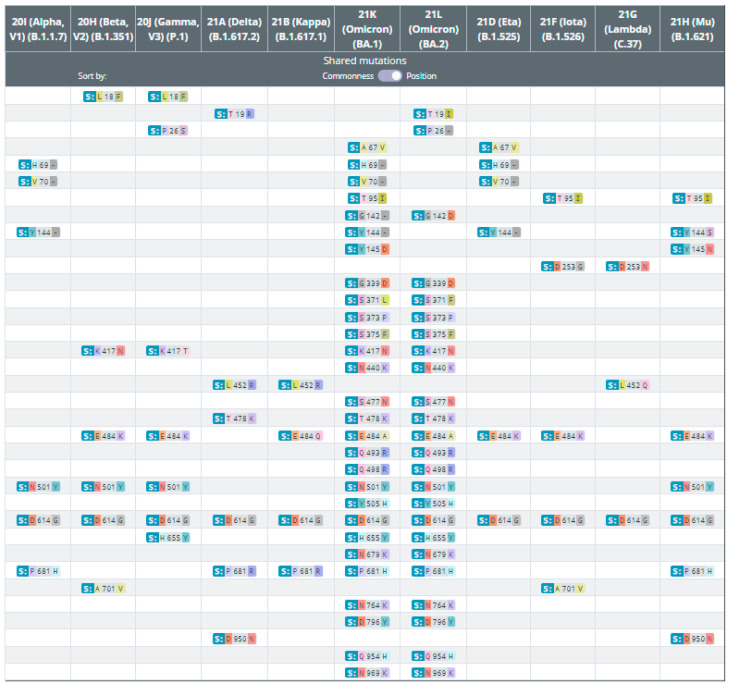
Omicron shares many mutations with four other VOCs, including Alpha, Beta, Gamma, and Delta.

News of the emergence of a new variant, called B.1.1.529 or Omicron, was reported in late November 2021, and triggered an urgent WHO meeting to critically assess the various aspects of the variant reported by South Africa [68] due to its many mutations. Among 50 new mutations, 30 were located in the sequence of the viral spike protein from the Omicron genome, which was the target of developed vaccines [69]. A comparison of the sequences from all of the variants of concern and variants of interest reveals that Omicron shares many mutations with four other VOCs, including Alpha, Beta, Gamma, and Delta (Figure 3. https://covariants.org/shared-mutations, accessed on 10 January 2022). Many of variants that emerged at the end of 2020 and beginning of 2021 share defining amino acid mutations. Some of these are mutations that are of interest to scientists. Table 1 displays the amino acid mutations shared by the variants below (top), and the other defining mutations of these variants (below). For example, P681H is shared among Omicron, Alpha and Delta), while D614G is the most common mutation from all trains, and is also present in the Omicron genome. Omicron mutations have implications for its functional biology, potentially adversely affecting vaccine effectiveness globally.

## 5. Variants under Monitoring (VUMs)

VUMs were detected through the genomic sequencing of different virus samples. These variants may carry similar mutations, which may impact the pathogenicity of VOCs. However, no strong evidence was observed for their impact on viral transmission/virulence. For example, C.12, discovered in South Africa in June 2021, may impact viral transmissibility and immune escape, which carried six spike mutations (D614G, E484K, H655Y, N501Y, N679K, Y449H). The B.1.1.318 variant with an unknown origin and C.36 with L452R (from Egypt) may also impact immune escape, but did not increase the disease transmissibility. In India, three VUM variants were found in spring 2021: B.1.617.2 with E484X; B.1.617.2 with Q613H; and B.1.617.2 with Q677H. However, none of these variants were proven to impact disease severity, transmissibility or immune escape. Even though additional VUMs were also identified in Italy (P.1 with P681H), the UK (B.1.617.2 with K417N, or in the Republic of the Congo (B.1.640), none of these were proven to be potentially dangerous variants: https://www.cdc.gov/coronavirus/2019-ncov/variants/variant-classifications.html, accessed on 10 January 2022, https://www.ecdc.europa.eu/en/COVID-19/variants-concern, accessed on 10 January 2022.

## 6. Current SARS-CoV-2 Vaccine Platforms

Since the outbreak of SARS-CoV-1, vaccine studies against coronaviruses became an important field. Several vaccines were evaluated, the majority of which were able to induce immunity by antibody production or T-cell responses in different animal models. Since the start of SARS-CoV-2 outbreak, studies were being conducted all around the world to develop strategies for safe vaccines. SARS-CoV-2 shared similarities with other Betacoronaviruses. The immune response of SARS-CoV-2 was found to be similar to the other previously discovered viruses, such as SARS-CoV-1 or MERS. All of these viruses could suppress the activation of the innate immune system, such as dendritic cells or interferon responses. The genomes of SARS-CoV-2 and SARS-CoV-1 shared 79% similarities with identical ACE2 receptors as a common target. However, the receptor affinity of SARS-CoV-2 was much higher than SARS-CoV-1. These similarities may help studies on vaccine development against SARS-CoV-2. These vaccines could be based on different platforms, including inactivated/attenuated viruses, viral proteins, virus-like particles, viral DNA/RNA or nanoparticles [74,75,76]. Studies on vaccines to protect against SARS-CoV-2 started in 2020, and the majority of these vaccines were developed to target the S-protein of the virus, since it was the key protein of viral entry to host cells. Vaccines that target the S-protein should enhance the production of antibodies and prevent viral entry. Animal experiments suggested that immunization against the RBD domain could protect against SARS-CoV-1 or MERS entry and induce the production of neutralizing antibodies. These strategies may suggest that the S1 subunit of the SARS-CoV-2 spike protein could also enhance IgG production and impact viral neutralization. However, S-protein-related vaccine development may be challenging, since neutralizing antibodies could have the risk that secondary SARS-CoV-2 infections may result in more severe disease phenotypes due to antibody-dependent enhancement (ADE). ADE could be caused by a high degree of virus replication. It may occur in respiratory infections, and be associated with respiratory diseases, such as SARS-CoV-1 or MERS. [77,78]. Furthermore, research should be performed to prevent or minimize the risk of other vaccine-related immune reactions, such as autoimmunity reactions, vaccine-induced disease enhancement, and T-cell-related immunopathology [79]. Studies on animal models should be important for understanding the protective mechanisms of vaccine candidates. The ideal animal models should clearly reflect human pathomechanisms and should also predict the immune responses of different vaccine candidates. Several mouse models were investigated against SARS-CoV-1, SARS-CoV-2 or MERS, such as BALB/c, C57BL/6, RAG1−/− or 129SvEv mice [79]. The issue with animal models was that a full replication of the disease/protection mechanisms in humans could not be achieved. Controlled human infection models (CHIM) were also used in vaccine development, which could speed up the researching and testing of the efficacy of potential drug or vaccine candidates. The issue with CHIM studies could be that volunteers were at risk of virus-related complications [80].

**Figure 4:**
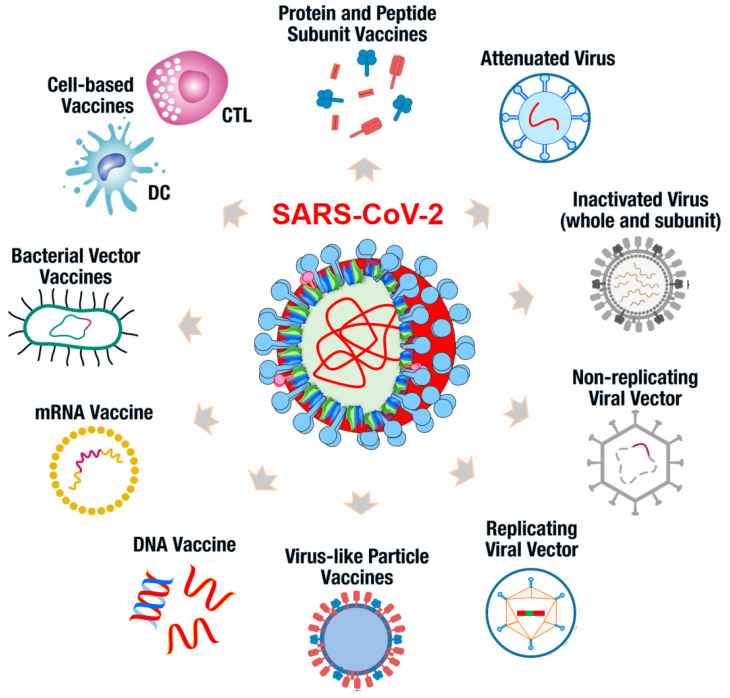
Vaccine platforms used for SARS-CoV-2 vaccine development.

The majority of vaccines against MERS and SARS-CoV-1 were based on the inactivated or live attenuated viruses, virus-like particles (VLP), nucleic acids (DNA or RNA) or proteins (Figure 4). All of these platforms had their benefits and disadvantages [74,81]. Table 2 shows the benefits and disadvantages of different vaccine platforms [79]. The approved vaccines were described to be generally safe since they were approved by clinical and pre-clinical studies. The probability of side effects, such as the neurological, articular, and autoimmune effects of vaccines, was reported to be rare. The issue with currently available SARS-CoV-2 vaccines was the absence of an assessment of their long-term side effects, which remain unclear [82].

## 7. Potential Mixing, Matching SARS-CoV-2 Doses and Booster Vaccines

As mentioned before, multiple vaccine platforms are available for SARS-CoV-2, and several vaccines are currently under development. The challenge is administering the appropriate vaccines to the right people at the right time. Since the highly infectious Delta and Omicron variants have appeared, the vaccination process should be sped up. The majority of current vaccine platforms require two doses with duration of 1–3 months between the first and second shots. Additionally, there are ongoing studies to identify the appropriate time for the third booster shot. Other factors for assessing time intervals and vaccine types for potential side effects are crucial. For example, the AstraZeneca vaccine may increase the risk of side effects in younger generations, such as thrombocytopenia, which changed the vaccination strategies and targeting of AstraZeneca shots for older populations. In several countries, after the first AstraZeneca vaccination, the mRNA vaccine (Pfizer) was initially suggested as the second dose. Even though no definite study was performed on heterologous vaccination, the mRNA vaccine as a second dose after AstraZeneca may result in stronger cellular and humoral immune responses in comparison to homologous vaccinations [90]. Shaw et al. (2021) screened the effects of heterologous shots in comparison to homologous shots in several individuals over 50 years of age into the following four groups: homologous AstraZeneca, homologous Pfizer, heterologous Pfizer/Astra Zeneca and or AstraZeneca/Pfizer.

Common post-vaccine symptoms were fever, fatigue, muscle or joint aches, headache, and malaise in both homologous and heterologous shots. Some symptoms, such as headaches, muscle pain and malaise were stronger among heterologous vaccine cases. This study did not observe any case of post-vaccine-related hospitalization 48 h after vaccination. In addition, no case of thrombocytopenia appeared among the examined cases. This study revealed that heterogeneous vaccination could have short-term disadvantages, and further studies are needed to determine their safety [91,92].

Canada’s National Advisory Committee on Immunization (NACI) initially did not recommend mixing and matching the AstraZeneca vaccine with Pfizer or Moderna shots. However, after the possible risk of blood clots from AstraZeneca, and due to limited supplies, NACI updated the advice on mixing and matching vaccines. Based on several current studies, no serious side effects appeared in adults who received second mRNA vaccines after first dose of AstraZeneca [93]. Hence, Schmidt et al. recommended the combination of the vector/mRNA vaccines since they may result in a higher anti-viral activity. The heterologous use of mRNA and vector-based vaccines resulted in a generation of spike-specific IgG and CD4/CD8 T-cell expressions. The mRNA vaccine as a second shot after vector-based vaccination is currently being recommended in Germany [94]. Borobia et al. (2021) also confirmed that combining vector- and mRNA-based vaccines could result in a stronger antiviral effect with a manageable reactogenicity [90]. Pozzetto et al. (2021) suggested that combing AstraZeneca and Pfizer vaccines could also provide better protection against viral infections in comparison with homologous Pfizer shots. The combination of two different anti-spike antibody responses may result in as higher degree of neutralization activity against any kind of SARS-CoV-2 variants. Heterologous vaccine shots may result in stronger immune responses. A stronger neutralization activity was associated with more frequent memory B-cell activation against the virus. The IgG responses were weaker than the homologous vaccines, but T-cell activation was higher [95]. Combining AstraZeneca and mRNA-based vaccines was suggested to increase protection against the SARS-CoV-2, including the Delta variant [96].

The current vaccines may provide only short-term protection against the SARS-CoV-2 virus. The third shot or booster shots may improve the protection against severe disease symptoms [97,98]. Booster shots are usually given after 5–6 months, followed by the second vaccination [92,99]. A booster shot may be needed to maintain protection against virus-related disease. Further studies are needed to screen the long-term effects of booster shots and to find out how long the effect of booster shots lasts [13]. The emergence of the new Omicron variant caused an urgency to speed up the inoculation of third doses all around the world. In the ideal case, boosters could possibly maintain protection against new virus variants, including Omicron [83,100]. Combining vaccines (for example, mRNA vaccines, followed by a protein vaccine as a booster shot) may also provide stronger protection against the Omicron variant [101,102]. Kuhlmann et al. (2022) revealed that patients with their third shot presented mild or moderate disease symptoms against the Omicron variant. Booster shots may provide good protection against severe forms of disease in case of future SARS-CoV-2 infections. However, this study only involved seven individuals who were relatively young and healthy. It is not yet known how vaccines and viruses could act in elderly populations and/or people with weaker immune systems. Additionally, the long-term effects of Omicron and the booster vaccination remain unclear [6].

## 8. Potential SARS-CoV-2 Variant Evolution Trend

Between December 2019 and 20 January 2022, 332,617,707 confirmed cases of SARS-CoV-2, including 5,551,314 deaths, were reported by the WHO (https://COVID19.who.int, assessed on 10 January 2022). Moreover, over 7.2 million SARS-CoV-2 genome variants were sequenced and shared via the Global Initiative on Sharing All Influenza Data (GISAID; https://gisaid.org, accessed on 10 January 2022), which reported many mutations, including >4100 mutations in the S-gene with 1200 missense mutations of amino acids and 187 in the RBD of the S-protein [103].

**Figure 5:**
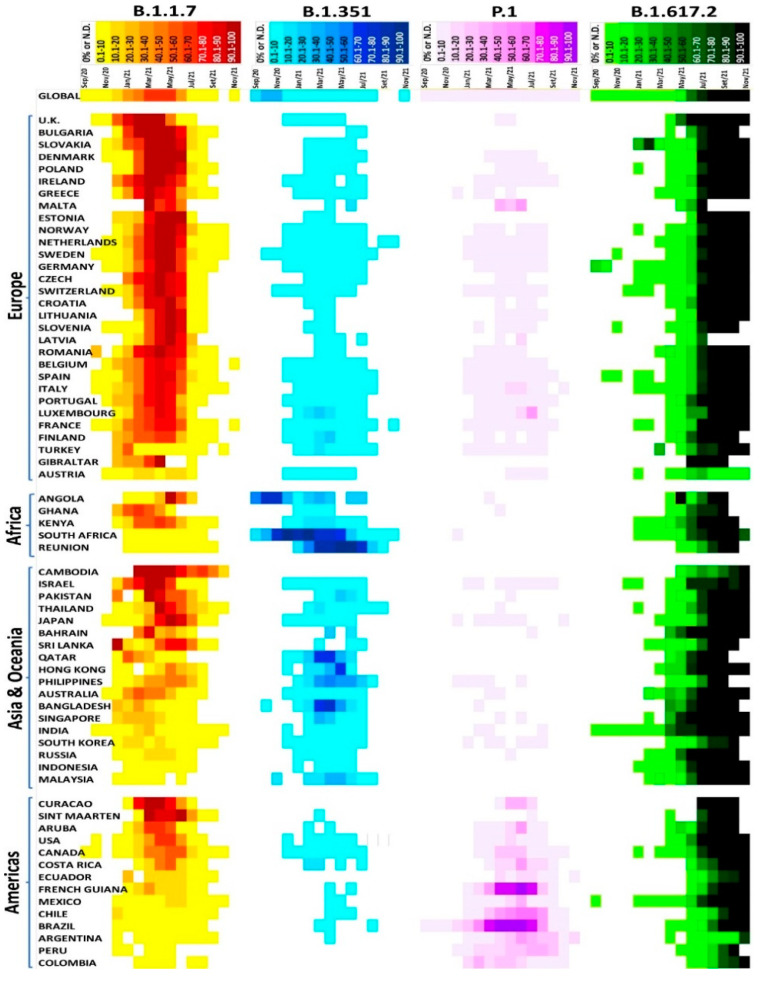
SARS-CoV–2 variants of concern distributed in several countries [104].

Remarkably, a recent study revealed the distribution of the SARS-CoV-2 variants of concerns in several countries, providing a general overview of specific variants globally [104]. For example, the B.1.1.7 variant rapidly spread to several countries across all continents, while the B.1.351 variant emerged in South Africa in August 2020, rapidly disseminating worldwide, reaching 111 countries, as of 2 September 2021 (Figure 5) [104].

All viruses accumulated mutations during their evolution. The speed of mutation accumulations and the outcomes of transmission and disease symptoms in the host may depend on many factors, such as rate of mutations, immunity, the presence of other viral factors and viral dynamics inside the host or between different individuals. SARS-CoV-2 may have a lower mutation rate in comparison to other RNA viruses, such as HIV virus or influenza. The viruses of SARS-CoV-2 that were VOCs exhibited twice as many mutations in comparison to non-VOCs. VOCs may accumulate several mutations in spike regions, especially in the receptor-binding domain. These variants could facilitate rapid transmission, ACE2–virus interactions or immune escape. Viral evolution was well-documented in immunocompromised patients, who maintained a higher viral load for longer periods. Sequencing the viruses revealed rapid changes, and the natural selection seemed to be faster in individuals with weaker immune systems [105,106,107,108,109]. As mentioned early, the reported new variants showed that research on the possible evolution of different viruses (including SARS-CoV-2) is essential. In many efforts, to predict future evolutionary maneuvers of SARS-CoV-2, current studies revealed mutations, which may enable the virus to be more resistant to natural human immunity, vaccines and antibody therapies.

## 9. Conclusions and Perspectives

During the last two years of the SARS-CoV-2 outbreak, different virus variants were reported to dominate distinct infection waves. These included the Alpha, Delta or Omicron variants, which overwhelmed the second, third and fourth waves globally. These variants came to prominence through the founder effect and advantageous selections, favoring rapid transmissions and allowing Alpha, Delta, and then Omicron to out-compete previous variants. In addition, the circulated Beta and Gamma variants in South Africa and Brazil, respectively, may have had a fitness advantage due to their distant antigenicity from the first wave of the virus and were able to re-infect people with higher efficiency.

Several different processes could have led to changes or alterations in mutation/variant frequencies, and changes were not always observed in variant frequency from the action of the natural selection. It may be difficult to estimate with certainty which novel viral variants could cause a high degree of infections and the kind of phenotypes that they may cause in large populations. The simultaneous screening of different data, such as a viral genome analysis, as well as the latest trends in epidemiology and quantifiable viral features, are essential to predict, determining whether an upcoming variant has a chance to become a VOC. It is important to note that the larger infestations of the virus in circulation will increase the risk of creating a greater number of variants. Genomic surveillance alone may not be enough since the phenotypes could not be predicted unambiguously. Hence, it is recommended that laboratory-based studies are conducted to predict the putative future evolution of currently existing virus variants and further interpret genomic surveillance, which would be crucial for defending future outbreaks.

The approved SARS-CoV-2 vaccines, or those in development, are expected to provide an effective defense against current and new virus variants without any autoimmune reaction or other side effects. Vaccines should induce an extensive immune response by activating different immune cells and producing appropriate amounts of antibodies to also protect people against possible novel VOCs. In the event that a vaccine is less effective against one or more variants, activated innate immunity may provide the first line of defense, and the fast assessment of the safety and effectivity of new vaccines against new variants would be streamlined. Even though currently developed vaccines may be effective in scaling down transmission and hospitalization, the research on long-term mucosal immunity in vaccinated individuals should be necessary. This could also lower the risk of viral transmission and variant selection from individuals who were inoculated against virus. SARS-CoV-2 may activate a broad scale of immune response. For example, virus-neutralizing antibodies could target specific sites on the surface of viral proteins. T cells may be able to recognize several viral peptide fragments, which may also be conserved between different VOCs or VOIs. The T-cell against SARS-CoV-2 response may result in a lower risk of viral immune escape. The high degree of T-cell response may enforce the evolution of the virus. The Delta variant was an example of escaping T-cell related immunity in one HLA type, which was specific to Asia. It remained unclear, mutations towards the T cell epitopes would be required for escape or to cause decreases in T cell immunity, and whether there was a hierarchy of crucial T cell epitopes. Combination therapy could provide a way to minimize viral evasion, typically involving two or more drugs or drugs and/or combination of targeting mAbs towards different viral functions, creating a higher barrier to create the resistance against viral evolution. However, the mass administration of combination therapy would be an even harder strategy to achieve.

The main goal is to stop the viral spread by cutting off the source of transmission. Restrictions and safety actions, such as washing hands; wearing masks, especially in crowded areas; maintaining social distancing; and avoiding crowds may be effective against novel VOCs or VOIs, since they could lower viral transmission. Additionally, these measures could also decrease the rate of viral mutations. Speeding up vaccine production and inoculations is also a crucial way to protect against current and potential future viral variants. Vaccination priority should be provided for high-risk individuals for better global protection and to reduce the possibility of transmission. Additionally, equal access of vaccines for everyone should be essential, since high vaccination rates would reduce viral circulation as well as its mutation ratio. Vaccines are playing a central role in the prevention of SARS-CoV-2, and their impact on human public health is confirmed (such as milder/no symptoms in case of infection and reduced hospitalization). Vaccination should be administered even if vaccines may not provide full protection against certain viral variants. Vaccine development should also continue to develop for a better protection against putative future variants.

## Figures and Tables

**Table 1:** VOC or VOI variants of SARS-CoV-2, based on the World Health Organization.

Virus Variant	Origin	Spike Mutations	Other Mutations	VOC/VOI	Properties of Significant Mutation	Refs
SARS-Wuhan	China	D614G	ORF1a: P323L, UTR: g.241C > T, g.23403A > G	VOC/ former VOC	D614G: enables rapid viral spreading	[11]
VOC 202012/01 or B.1.1.7 or Alpha variant	UK	A570D, D614G, D1118H, H69del, N501Y, P681H, S982A, T716I, del_V69-70, Y145del	N: D3L, S235F ORF8: Q27X, R52I, Y73C ORF1a: G465S, A890D, I1412T, T183I, del_S106, del_G107, del_F108, P323L, G204R, R203K	VOC/ former VOC	N501Y: increases of viral biding to ACE2 receptor P681H: enhances viral entering to host del_V69-70: putative immune escape variant	[70,71]
GH501Y.V2 or B.1.351 or Beta variant	South Africa	D614G, D80A, D215G, E484K, N501Y, A701V, L18F, R246I, K417N, del_aa242-244	E: P71L N: T205I ORF1ab: K1655N, del_aa3675-3677, T265I, H2799Y, P4715L	VOC	E484K and N500Y: enhance the ACE2 binding to virus K417N: enhances further viral binding to ACE2 All of them: immune escape	[20]
P1, B.1.1.28; GR/501Y.V3 or Gamma variant	Brazil	L18F, T20N, P26S, D138Y, R190S, K417T, E484K, N501Y H655Y, T1027I, D614G	ORF1ab: N: P80R, R203K ORF3a: S235P ORF8: E92K ORF1ab: P323L, K977Q, del_aa106-107 UTR: g. 733T > C, g. ins28263AACA, g. 28877A > T, c.28878G > C, c.12778C > T, del11288-11296	VOC	N501Y and E484K: Virus-ACE2 interaction enhancement K417T: immune escape	[72]
B.1.617 or Delta variant	India	T19R, del_aa157-158, L452R, T478K, D614G, P681R D950N	ORF1a: A1306S, P2046L, P2287S, V2930L, T3255I, T3646A ORF1b: P314L, G662S, P1000L, A1918V ORF3a: S26L M: I82T ORF7a: V82A, T120I ORF7b: T40I ORF8→del119/120 N: D63G, R203M, D377Y	VOC	L452R and T478K: immune escape P681R: enhances viral ability to enter to host, increases viral load	[32,33]
B.1.1.529 or Omicron variant	South Africa	A67V, del_V69-70, T95I, G142D/del_aa143-145, Δ211/L212I, ins_214EPE, G339D, S371L, S373P, S375F, K417N, N440K, G446S, S477N, T478K, E484A, Q493R, G496S, Q498R, N501Y, Y505H, T547K, D614G, H655Y, N679K, P681H, N764K, D796Y, N856K, Q954H, N969K, and L981F	ORF1a: del_L3674-S3675 and G3676 N: R203K G204R	VOC	N501Y, H655Y, P681H, N679K, and D614G: high transmissibility Q498R and N501Y: higher ACE2 binding K417N and T478K: ability for immune escape	[39]
B.1.427/B.1.429, or Epsilon variant	USA (California)	B.1.427: L452R, D614G; B.1.429: S13I, W152C, L452R, D614G	ORF1ab: T85I, I64V, P323L, D260Y ORF3a: Q57H N: T205I	Former VOI	L452R: enhance viral infectivity, fusion and viral binding to ACE2	[45]
C.37 or Lambda	Peru	G75V, T76I, D253N, L452Q, F490S, D614G, T859N	ORF1ab: del3675 to 3677, T428I, P323L N: P13L, P10S, R203K, R204G, R214C, 246_253delinsN	VOI	L452Q, F490S: may enhance viral affinity to host	[48]
B.1.617.1 or Kappa	India	T95I, G142D, E154K, L452R, E484Q, D614G, P681R, Q1071H	ORF1ab: T749I, T77A, P323L, M429I, K259R ORF3: S26L Orf7a: V82A N: R203M, N377I	Former VOI	L452R and E484Q: may enhance viral affinity to host P681R: Influence viral entry into host	[52]
B.1.526 or Iota	USA (New York)	L5F, T95I, D253G, S477N, L452R, E484K, D614G, A701V	Orf1ab: T85I, L438P, del_aa106-108, Q57H Orf3: P199L N: M231I	Former VOI	L452R, S477N and E484K: may enhance viral affinity to host	[56]
B.1.525 or Eta	USA (New York	A67V, del_aa69-70, del_aa144, E484K, D614G, Q677H, F888L	ORF1ab: T1189I, P323F E: L21F M: I82T N: del_aa3, A12G, T205I	Former VOI	Q677H: enhances viral transmission del_aa144: immune escape F888L: putative virulence functions	[55]
B.1.621 or Mu	Columbia	del_aa69-70, Y144T, Y145S, ins_146N, R346K, E484K, N501Y, D614G, P681H, and D950N	ORF1ab: T237A, T720I, T492I, Q160R ORF3a: Q57H, del_aa256-257 ORF8: T11K, P38S N: T205I	VOI	del_aa69-70, E484K and N501Y: impact the antibody neutralization, immune escape ins146N: ACE2 binding	[73]
P.3 or Theta	Philippines	del_aa141-143, E484K, N501Y, P681H, D614G, H1101Y, E1092K, V1176F, G593G and S875S	ORF1ab: D736G, L438P, L71F, A368V ORF8: K2Q N: R203K, G204R	Former VOI	E484K, N501Y, P681H: enhance viral infectivity, Viral ACE2 binding del_aa141-143: immune escape	[62]
P.2 or Zeta	Brazil	E484K, F565L, D614G, V1167F	ORF1ab: L205V, L71F, P323L N: A119S	Former VOI	E484K and D614G: rapid spread, immune escape	[63]
AY.4.2, B.1.617.2 or Delta plus	India, UK	Delta variant mutations + T95I, A222V, Y145H G142D, R158G, and K417N	Delta variant mutations + ORF1A: A1146T, P1604L, A3209V, V3718S, and T3750I).	VOI	Y145H and A222V: enables viral penetration to blood cells K417T: immune escape	[64]

**Table 2:** SARS-CoV-2 vaccine platforms.

Vaccine Platform	Description	Benefits	Disadvantages	Examples of SARS-CoV-2 Vaccines	Refs
Protein subunit	Use specific viral part (spike protein) for appropriate immune response	Safe, stable, lower risk for autoimmunity or other side effects	Production may be costly, difficult to purify, and growing pathogens may be difficult; long-term protection may be doubtful	Novavax	[83,84]
Inactivated virus	Vaccine contains killed virus, which induces immune system	Successful platform against different viruses, may speed up research	Production may be costly, difficult to purify, and growing pathogens may be difficult	Janssen/Johnson & Johnson, AstraZeneca, SinoVac. BBV152	[85]
Live attenuated virus	Uses alive, but weakened viruses	May induce a broad degree of immune responses; human systems may adapt effectively	Vaccine is not approved yet; under development	COVI-VAC	[86]
DNA	Two or three dose plasmid DNA, which encodes the viral S-protein (or N-protein), with potential signal peptide	Easy to produce, Can be produced in large amount No pathogens are used More stable than RNA vaccines	Development is in early stage; not tested in humans DNA may insert into human DNA Risk of autoimmunity	ZyCoV-D, AG0302-COVID19	[87,88]
RNA	Two doses of vaccine, using the synthetic viral RNA, inducing the cells producing S-protein, resulting in antibody production against SARS-CoV-2	Easy to produce, Able to produce in large quantity No pathogen, generally safe No risk for inserting into human genome	Not too stable, need to be kept at low temperature (−60 °C) Risk for autoimmunity	Pfizer/BioNTech Moderna	[88,89]

## Data Availability

Not applicable.
